# Low SAA4 gene expression is associated with advanced HCC stage and a poor prognosis

**DOI:** 10.1007/s10238-023-01279-8

**Published:** 2024-02-01

**Authors:** Shilong Li, Dejun Kong, Weiqi Zhang, Yan Li, Hao Wang, Ruining Yang, Qian Sun, Zhenglu Wang, Zhongwei Zhang

**Affiliations:** 1 Department of Gastrointestinal Surgery, Shaoxing Second Hospital, Zhejiang, China; 2 Biological Sample Resource Sharing Center, Tianjin First Central Hospital, Tianjin, China; 3 School of Medicine, Nankai University, Tianjin, China; 4 The First Central Clinical School, Tianjin Medical University, Tianjin, China

**Keywords:** Hepatocellular carcinoma (HCC), Biomarker, SAA4, Alpha-fetoprotein (AFP)

## Abstract

At present, although there are tumor markers for hepatocellular carcinoma (HCC), markers with better predictive efficiency are needed. SAA4 gene expression in liver tumor and paracancerous tissues was analyzed using The Cancer Genome Atlas database. The differentially expressed genes (DEGs) were analyzed and visualized by heatmap and volcano plot. Survival analysis was performed based on SAA4 expression. SAA4 expression was compared in patients grouped based on clinicopathological features, and gene set enrichment analysis (GSEA) was conducted. Immunohistochemical staining was used to verify the SAA4 protein staining intensity from The Human Protein Atlas database and our center’s samples. The diagnostic value of SAA4 for HCC was evaluated by receiver operating characteristic curves. SAA4 was expressed at low levels in HCC tissues, and low SAA4 expression was associated with a poor prognosis in HCC. In addition, SAA4 expression decreased with HCC progression. There were 188 upregulated DEGs and 1551 downregulated DEGs between the high and low SAA4 expression groups. Complement and coagulation cascades, fatty acid metabolism, and ECM receptor interaction were significantly enriched in the GSEA. SAA4 had good predictive efficacy for HCC and even early HCC and was superior to AFP. In general, low SAA4 expression was associated with advanced HCC stage and a poor prognosis. In addition, SAA4 may be helpful for the diagnosis of early HCC and may become a novel tumor marker with good predictive power for HCC.

**Supplementary Information:**

The online version contains supplementary material available at 10.1007/s10238-023-01279-8.

## Introduction

Primary liver cancer (LC) is one of the most common malignant tumors in the world, with the sixth highest incidence and third highest mortality rate [1]. Primary LC is the fourth most common malignancy in China, with a mortality rate second only to that of lung cancer, partly due to chronic infection with hepatitis B virus (HBV) [2]. The vast majority of LC cases are hepatocellular carcinoma (HCC). In recent years, the mortality rate of HCC has increased by approximately 2% annually, which is closely related to the fact that HCC is usually diagnosed at an advanced stage and there are no effective strategies for advanced HCC [3]. Hence, early screening or diagnosis of HCC is crucial for improving prognosis.

Currently, serological alpha-fetoprotein (AFP) detection and ultrasonography (US) examination are the main means for early HCC monitoring [4, 5]. Nevertheless, the ability of ultrasound and AFP to screen for early-onset HCC remains unsatisfactory [6, 7]. With the development of imaging technology, including magnetic resonance imaging (MRI) and computed tomography (CT), the sensitivity and specificity of the diagnosis of early HCC have been improved [8–10]. However, due to the characteristics of high cost and radiation, MRI and CT are not suitable for the early screening of HCC. Therefore, it is necessary to explore novel biomarkers with high sensitivity and specificity for the early detection of HCC.

The serum amyloid A protein (SAA) family consists of acute phase SAA (A-SAA), including SAA1 and SAA2, SAA3, and constitutive SAA (C-SAA), also known as SAA4 [11–13]. A-SAA is designated acute phase SAA, which is significantly elevated during the acute response phase. SAA3, a pseudogene, is expressed in mice but not humans [12]. In addition, constitutive SAA4 is synthesized mainly in the liver, accounting for approximately 90% of the total SAA proteins in homeostasis [14]. SAA proteins have long been recognized as highly sensitive biomarkers associated with inflammation [15–17]. Studies have shown that inflammation is closely related to cancer progression, and cancer-related inflammation is primarily related to the local immune response at the tumor site, which usually precedes tumor development and promotes tumor progression [18]. However, it is unclear whether SAA4 plays a critical role in the progression of tumor such as HCC.

In this study, the differential expression of SAA4 in liver tumor and paracancerous tissues and the relationship between SAA4 expression and HCC stage and prognosis were investigated. In addition, the predictive value of SAA4 for HCC and even early-onset HCC was also discussed and verified.

## Materials and methods

### Collection of information on HCC patients from the TCGA database

The gene transcriptome expression and clinical profiles of 375 HCC patients were downloaded from the TCGA database (https://portal.gdc.cancer.gov/). In addition, the expression of SAA4 in HCC was also extracted and analyzed.

### Survival analysis based on the expression of SAA4 in HCC

The Kaplan‒Meier survival curves were plotted according to the expression of SAA4 based on the data from the TCGA database and Kaplan‒Meier (K‒M) plotter database. Furthermore, the survival times of HCC patients were also compared according to log-rank test.

### Differential expression analysis of SAA4 and correlation between SAA4 and clinicopathological variables

The Mann‒Whitney test and Wilcoxon paired test were conducted to compare the expression of SAA4 between tumor and normal tissues from the TCGA database. The Mann‒Whitney test and Kruskal‒Wallis test were also applied to compare the expression of SAA4 in the different groups of clinicopathological variables.

### DEG analysis, volcano plot and heatmap

The DEGs between the high- and low- expression group of SAA4 were screened with the thresholds of |logFC|> 1 and FDR < 0.05 and visualized by volcano plot. In addition, the heatmap was employed to illustrate the DEGs using the “pheatmaps” R packages.

### Gene set enrichment analysis (GSEA)

Kyoto Encyclopedia of Genes and Genomes (KEGG) and Gene Ontology (GO) gene sets database were downloaded from Signature Database (MsigDB, http://software.broadinstitute.org/gsea/downloads.jsp). GSEA was conducted by using the “clusterProfiler” package in R, and gene sets with |ES|> 0.5 and *p* value < 0.05 were identified.

### The receiver operating characteristic (ROC) curves

ROC curve analysis is an effective method to evaluate the performance of diagnostic tests. The area under the ROC curve was calculated and applied to investigate and compare the predictive efficacy of SAA4 and AFP in HCC.

### Immunohistochemical (IHC) staining

To investigate the differential expression of SAA4 between HCC and paired paracancerous tissues, the staining density difference was compared by the IHC staining results from The Human Protein Atlas (THPA, http://www.proteinatlas.org/). To further confirm this, a total of 40 pairs of tumor and paracancerous tissues were harvested from patients with HCC who underwent radical hepatectomy and were stored in the Biological Sample Resource Sharing Center. This study was approved by the Ethics Committee of Tianjin First Center Hospital (STEC-TFCH-2023-HM-2).

The tumor and paracancerous tissues embedded in formalin were cut into 5-μm slices for IHC staining. Briefly, the slices were dewaxed using a one-step dewaxing solution (Elabscience, E-CK-A032, Wuhan, China). Dewaxed slices were next subjected to antigen retrieval using sodium citrate solution (Solaribio, C1032, Beijing, China) in a microwave. After that, endogenous peroxidase was removed with 3% H_2_O_2_ and antigen blocking was performed with goat serum. Anti-human SAA4 antibody (Abcam, ab92540, 1:50, US) was added dropwise and incubated overnight at 4℃, and then, secondary antibody staining and DAB chromogenic reaction were performed (ZSGB-BIO, PV-9000, Beijing, China). Finally, images were analyzed using Image-Pro Plus, and the expression of SAA4 was quantified using the IOD index.

### Statistical analysis

R software (version 4.2.0) and GraphPad Prism 8 were used to perform statistical analysis and data visualization. The Chi-square test and Fisher’s exact test were employed for categorical variables, and Student’s t test was conducted for continuous variables. The correlation between SAA4 expression and clinicopathological features was also analyzed by the Mann‒Whitney test and Kruskal‒Wallis test. A *P* value < 0.05 was considered to indicate statistical significance.

## Results

### SAA4 is expressed at low levels in HCC

**Fig. 1:**
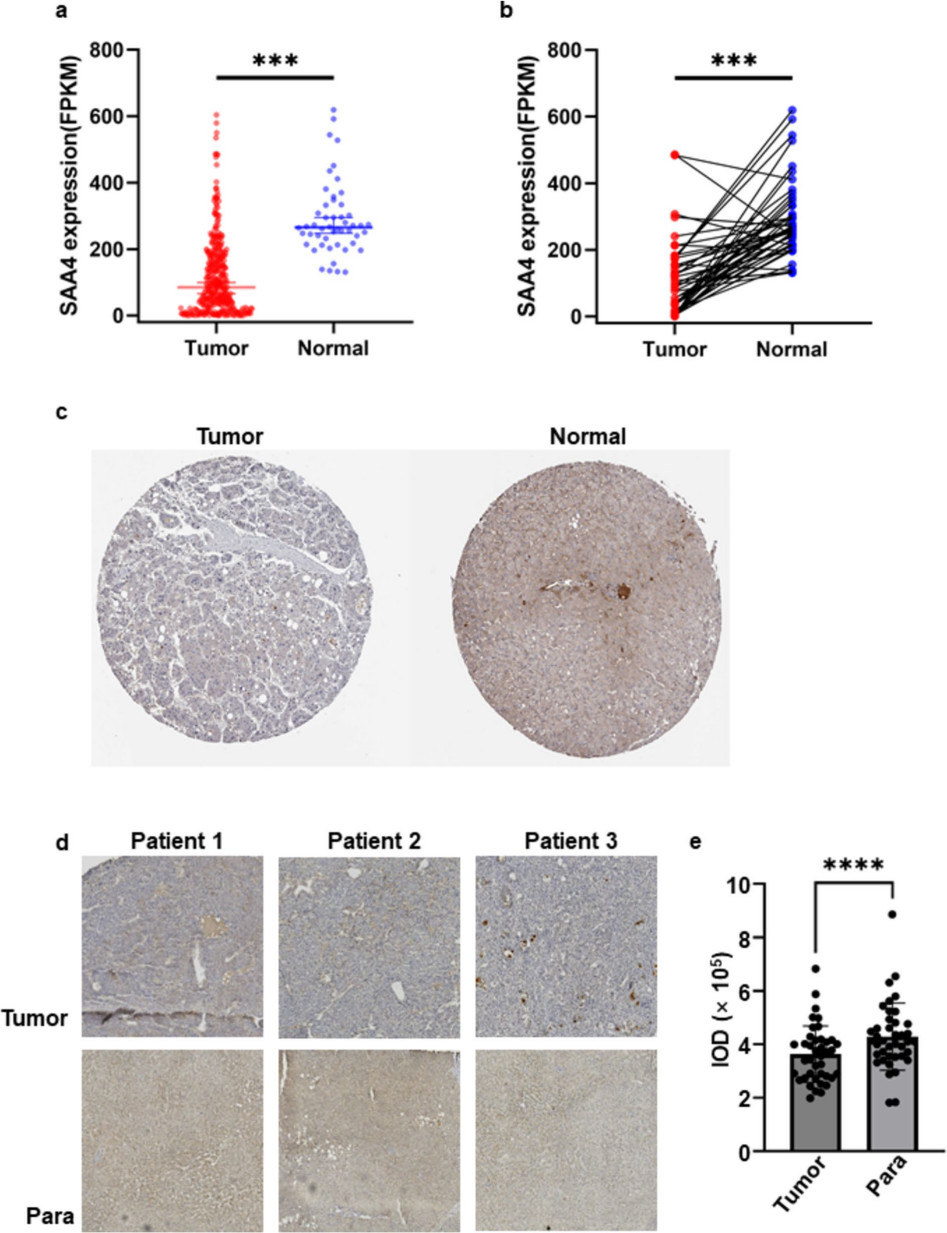
SAA4 expression in HCC tissues was significantly lower than that in normal tissues. **a** SAA4 was expressed at low levels in 375 HCC tissues compared with 50 normal tissues. **b** In the paired analysis, SAA4 expression was also significantly lower in tumor tissues than in normal tissues. **c** IHC staining results from the THPA database showed that the staining intensity of SAA4 protein in HCC was significantly weaker than that in normal tissues. **d** IHC staining results from our center showed that the staining intensity of SAA4 protein in HCC tissues was significantly weaker than that in normal tissues, and the IOD values were evaluated by semiquantitative analysis (**e**). FPKM: fragments per kilobase of exon model per million mapped fragments; ***P value < 0.001; ****P value < 0.0001

A total of 375 HCC patients from the TCGA database and 40 HCC patients from our center were enrolled. In the analysis of the entire TCGA cohort, SAA4 was expressed at low levels in 375 HCC tissues compared with 50 normal tissues (Fig. 1a). In addition, the expression of SAA4 was also significantly lower in tumor tissues than in normal tissues in the paired analysis (Fig. 1b).

In the IHC staining analysis, the SAA4 protein expression in normal tissues was higher than that in tumor tissues according to the results from the THPA (Fig. 1c). To further verify the SAA4 expression difference, IHC staining was applied for the detection of the SAA4 protein in the tumor and paracancerous tissues from our center, which showed that the staining density of SAA4 in paracancerous tissues was stronger than that in tumor tissues (Fig. 1d, e, *P* < 0.001).

### Low expression of SAA4 is associated with the progression and poor differentiation of HCC

To investigate the role of SAA4 in the progression of HCC, correlation analysis between SAA4 expression and TNM stage, AJCC stage and grade was conducted. These results indicated that SAA4 expression was significantly different at different T stages, AJCC stages and histological grades, but no significant difference was observed in patients grouped by N stage (Figure S1a) or M stage (Figure S1b).

**Fig. 2:**
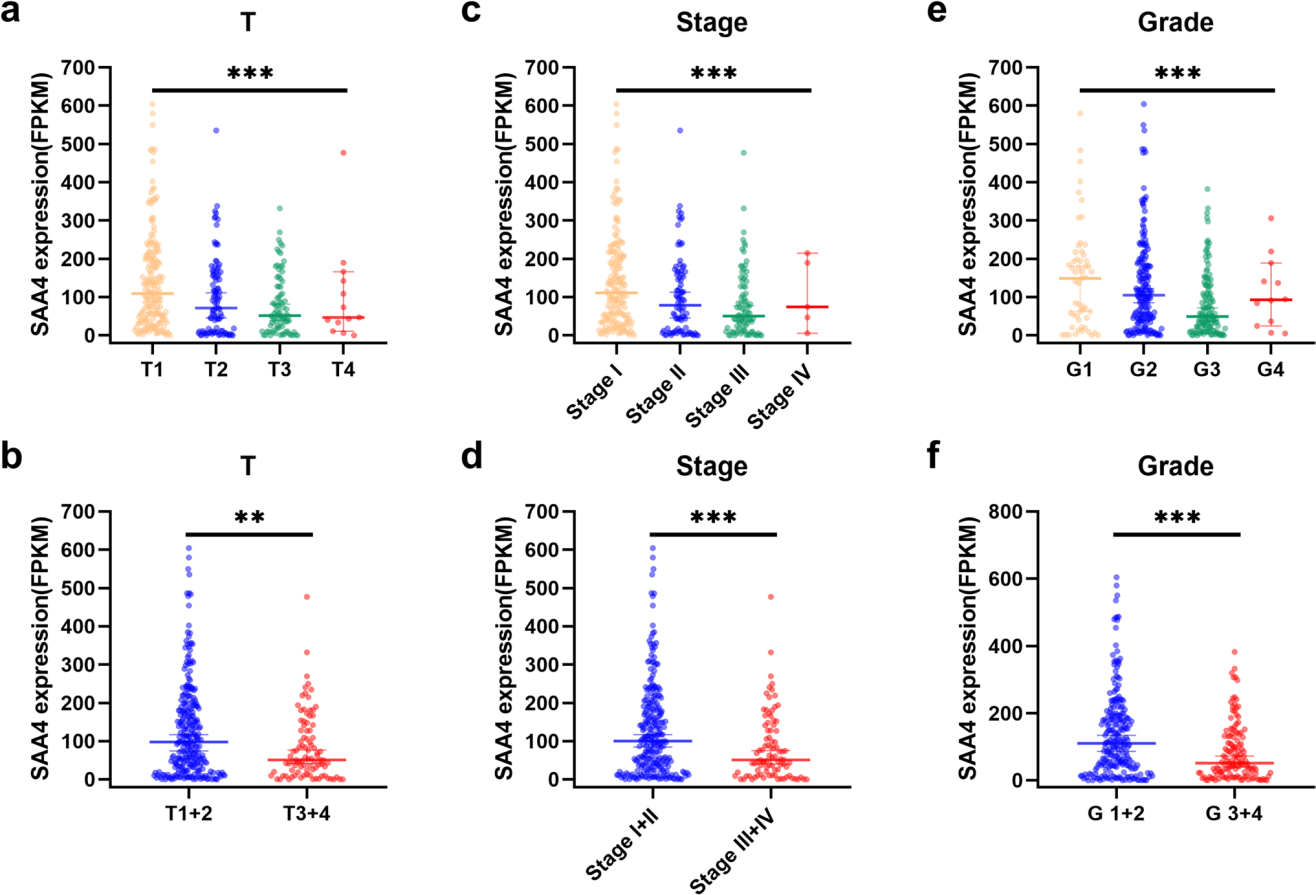
Low SAA4 expression was associated with HCC progression and poor differentiation. **a** SAA4 gene expression decreased with T stage progression. **b** SAA4 expression in the T1 and T2 stages was significantly higher than that in the T3 and T4 stages. **c** SAA4 expression was significantly different between the four stage groups. **d** SAA4 expression in stage I and stage II was significantly higher than that in stage III and stage IV. **e** SAA4 expression was significantly different between the four grade groups. **f** SAA4 expression in G1 and G2 was significantly higher than that in G3 and G4. FPKM: fragments per kilobase of exon model per million mapped fragments; **P value < 0.01; ***P value < 0.001

In terms of T stage, the expression of SAA4 decreased with the progression of T stage (Fig. 2a). In the T1 and T2 stages, the expression of SAA4 was significantly higher than that in the T3 and T4 stages (Fig. 2b). Although there were significant differences in the expression of SAA4 between different AJCC stages, the expression of SAA4 was not completely decreased with the increase in AJCC stage (Fig. 2c). At stage IV, the expression of SAA4 was not significantly decreased but was slightly increased compared with that in stage III. However, the small number of stage IV patients may have contributed to this result. To reduce the effects of this factor, the differences in SAA4 between stage I and II and stage III and IV were compared, which showed that the expression of SAA4 in stages I and II was significantly lower than that in stages III and IV (Fig. 2d). In addition, similar results were also observed in histological-grade correlation analysis, where the decrease in the expression of SAA4 was accompanied by an increase in histological grade (Fig. 2e, f). Taken together, these results suggest that low expression of SAA4 is significantly associated with the progression and poor differentiation of HCC.

### Low expression of SAA4 is associated with poor prognosis in HCC patients

**Fig. 3:**
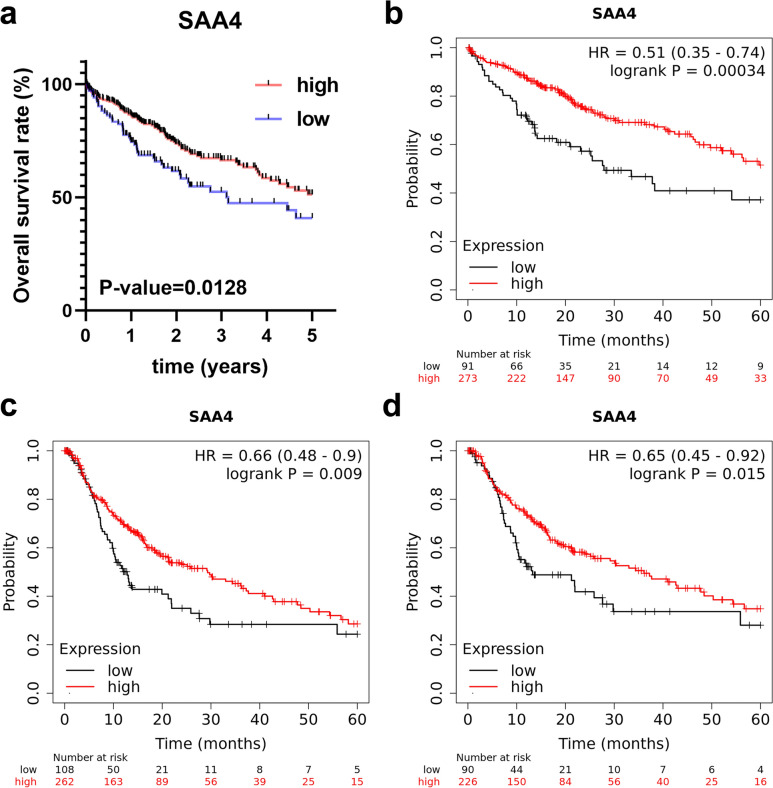
Low SAA4 expression was closely related to a poor prognosis in HCC. **a** The survival analysis results from TCGA database showed that 5-year OS of low-expression group was significantly worse than high-expression group. **b** The survival analysis results from K‒M plotter database showed that 5-year OS of low-expression group was also significantly worse than high-expression group. **c** The survival analysis results from K‒M plotter database showed that 5-year PFS of low-expression group was also significantly worse than high-expression group. **d** The survival analysis results from K‒M plotter database showed that 5-year RFS of low-expression group was also significantly worse than high-expression group. OS, overall survival; PFS, progression-free survival; RFS, recurrent-free survival; HR, hazard ratio

To further explore the effects of SAA4 on the prognosis of patients with HCC, survival analysis was carried out in the TCGA database and was verified in K‒M plotter database. In the TCGA database, HCC patients were divided into low- and high-expression groups. As shown in Fig. 3a, the 5-year overall survival (OS) of the high-expression group was significantly higher than that of the low-expression group. To further validate the effects of SAA4 on the outcomes of HCC patients, external validation based on K‒M plotter database was performed. Similarly, low expression of SAA4 was significantly associated with worse 5-year OS of HCC based on the K‒M plotter database (Fig. 3b). In addition, similar results were also observed for 5-year progression-free survival (PFS) and 5-year recurrence-free survival (RFS). The 5-year PFS (Fig. 3c) and 5-year RFS rates (Fig. 3d) of the high-expression group were significantly better than those of the low-expression group. Accordingly, HCC patients with low expression of SAA4 had a worse prognosis.

### DEGs, heatmap, volcano plot and GSEA

**Fig. 4:**
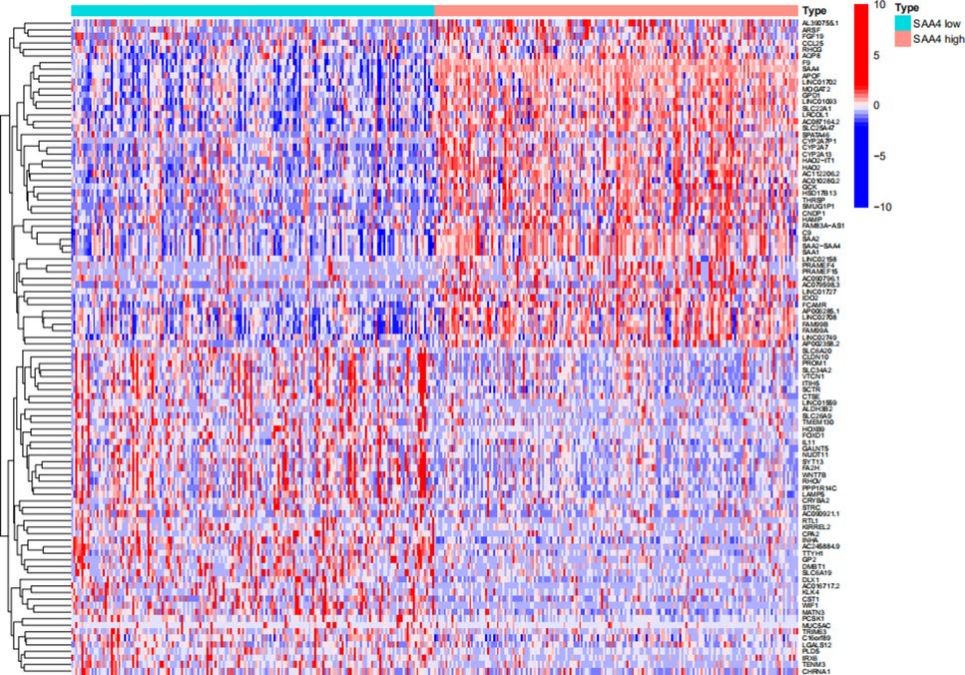
Comparison of differentially expressed gene profiles between SAA4 high- and low- expression groups in HCC. A heatmap was used to visualize differentially expressed genes between the high and low SAA4 groups

**Fig. 5:**
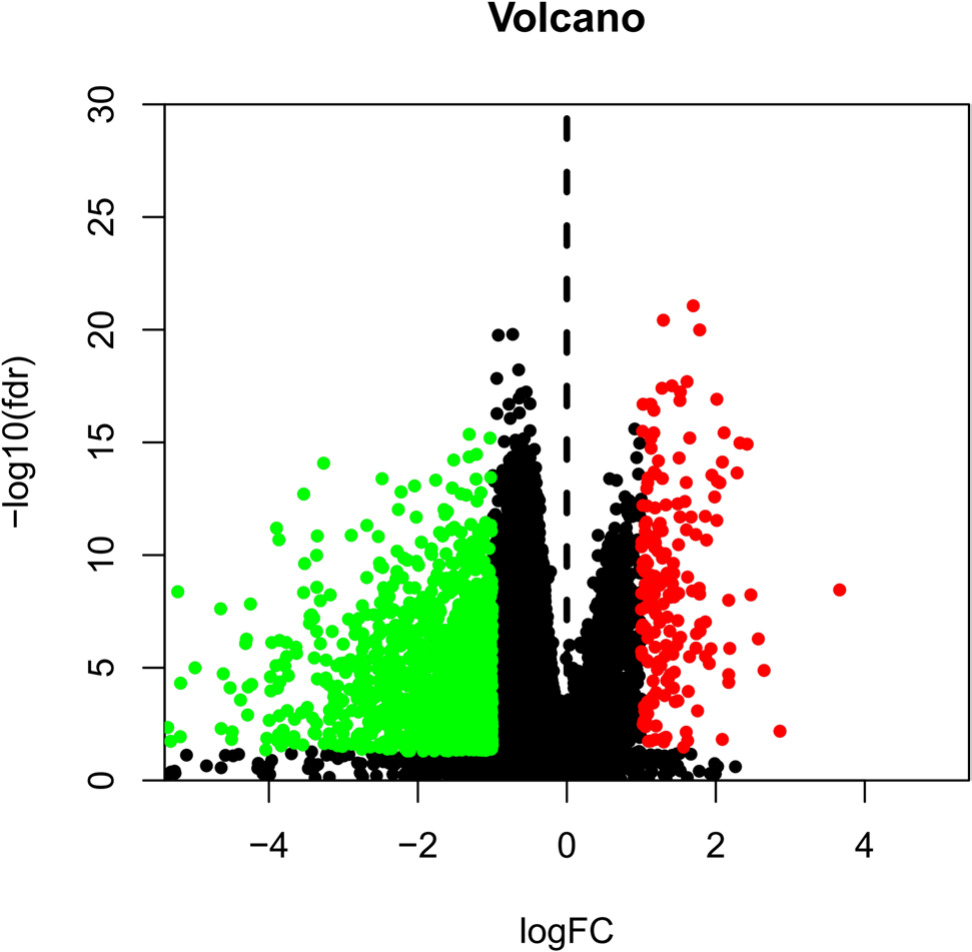
The differentially expressed genes between the high and low SAA4 expression groups in HCC. A volcano plot was used to visualize differentially expressed genes between the high and low SAA4 groups. FC, fold change; FDR, false discovery rate

To explore the underlying mechanisms of the effect of SAA4 on the progression of HCC, DEG, heatmap, volcano plot and GSEA analyses were conducted. There were 1551 genes with low expression and 188 genes with high expression between the SAA4 high- and low-expression groups. All the DEGs were identified and visualized using heatmap (Fig. 4) and volcano plot (Fig. 5).

**Fig. 6:**
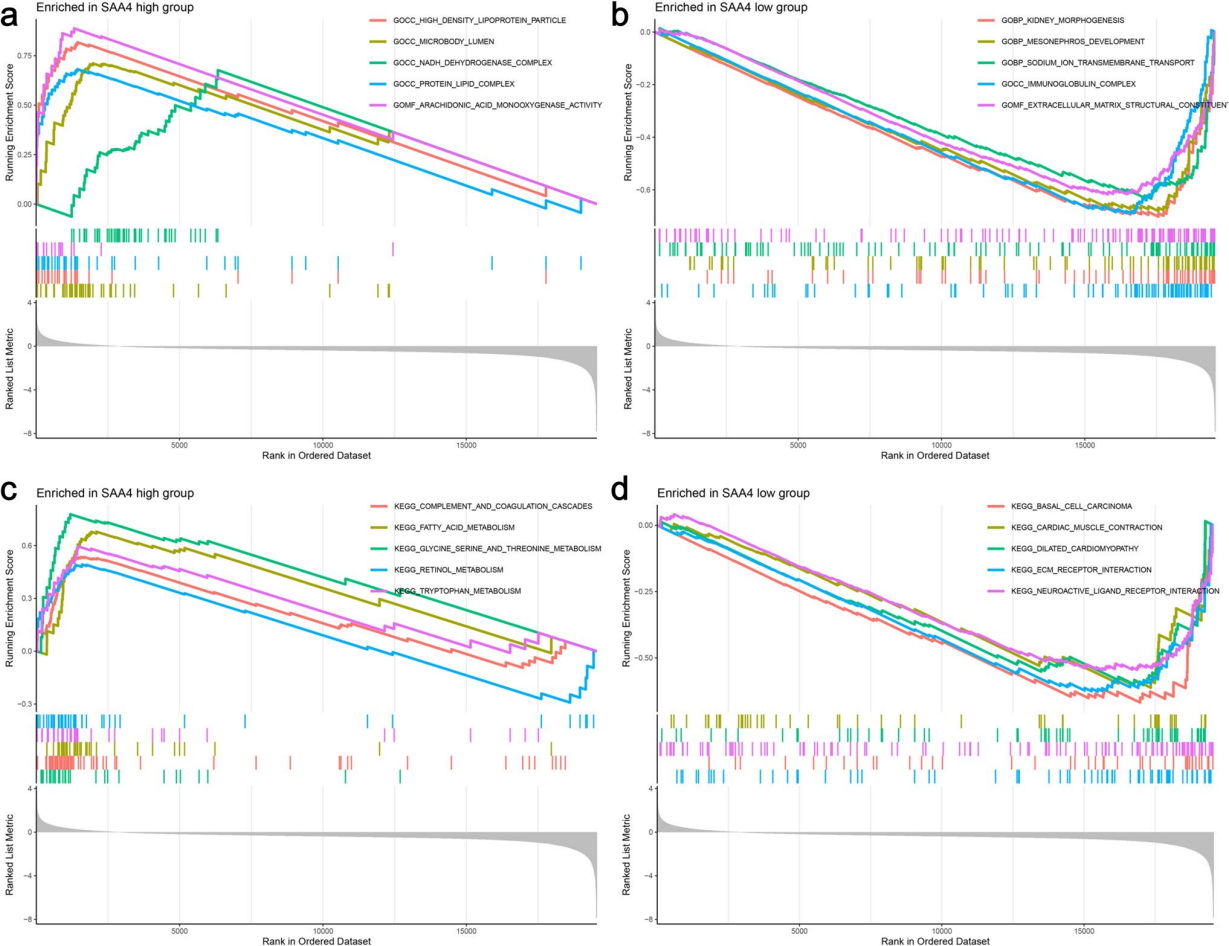
Gene set enrichment analysis (GSEA). **a** The top 5 GO terms in the high SAA4 expression group. **b** The top 5 GO terms in the low SAA4 expression group. **c** The top 5 KEGG terms in the high SAA4 expression group. d: The top 5 KEGG terms in the low SAA4 expression group

GSEA was employed to reveal the underlying mechanisms of SAA4 in the progression of HCC. As shown in Fig. 6a, the top 5 GO and KEGG terms in the high and low SAA4 expressions groups were identified. GO analysis results revealed that in the high-expression SAA4 group, the DEGs were mainly enriched in the following GO terms: high-density lipoprotein particle, microbody lumen, NADH dehydrogenase complex, protein lipid complex and arachidonic acid monooxygenase activity. In the low-expression SAA4 group (Fig. 6b), the DEGs were mainly enriched in the following terms: kidney morphogenesis, mesonephros development, sodium ion transmembrane transport, immunoglobulin complex and extracellular matrix structural constituent. In the KEGG analysis, the results indicated that in the high SAA4 expression group (Fig. 6c), the DEGs were mainly involved in complement and coagulation cascades, fatty acid metabolism, glycine serine and threonine metabolism, retinol metabolism and tryptophan metabolism. However, in the low SAA4 expression group (Fig. 6d), the DEGs were mainly involved in basal cell carcinoma, cardiac muscle contraction, dilated cardiomyopathy, ECM receptor interaction and neuroactive ligand receptor interaction. In this part, the DEGs between the high and low SAA4 groups were screened and visualized with heatmap and volcano plot, and the potential role of SAA4 in HCC was also investigated by GSEA.

### SAA4 has a higher predictive value than AFP for HCC

To investigate the predictive value of SAA4 for HCC and the complementary ability of SAA4 for AFP in predicting HCC, ROC curves were established, and corresponding AUC values were also calculated. Based on the optimal cutoff value of AFP, these HCC patients were divided into AFP-negative and AFP-positive groups. The cutoff value of AFP was 2.084, and its sensitivity and specificity for predicting HCC were 0.505 and 0.940, respectively. Similarly, the cutoff value of SAA4 was 195.737, and its sensitivity and specificity were 0.900 and 0.802, respectively. Then ROC curves were constructed.

**Fig. 7:**
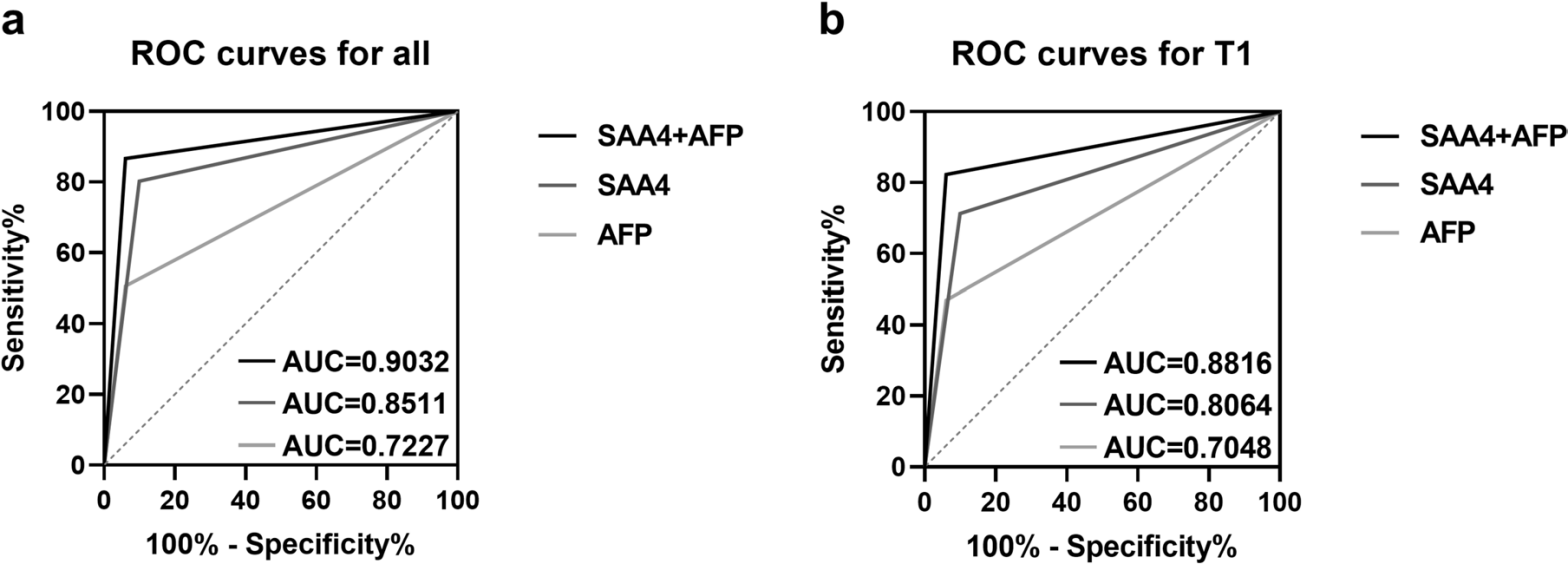
ROC curves were used to compare the predictive value of SAA4, AFP and the combination of SAA4 and AFP for HCC. **a** In the entire HCC cohort, the AUC value of SAA4 (AUC = 0.8511) was significantly higher than that of AFP (AUC = 0.7227), and the combination of SAA4 and AFP had better predictive efficacy (AUC = 0.9032). **b** In T1 stage HCC patients, the AUC value of SAA4 (AUC = 0.8064) was significantly higher than that of AFP (AUC = 0.7048), and the combination of SAA4 and AFP also had better predictive efficacy (AUC = 0.8816). AUC, area under the curve

As shown in Fig. 7a, the AUC value of SAA4 was 0.8511, which indicated that it is a valuable predictor for HCC. In addition, the AUC value of SAA4 was higher than that of AFP (AUC = 0.7227), which means that SAA4 is better for predicting HCC than AFP. Moreover, the combination of SAA4 and AFP had the best predictive performance for HCC; it was significantly better than AFP and SAA4 alone. The AUC value of the combination of SAA4 and AFP was 0.9032. Furthermore, to identify the predictive efficacy of SAA4 for early HCC, we also constructed ROC curves to evaluate the predictive efficiency of SAA4 for HCC with T1 stage. The result is shown in Fig. 7b; SAA4 also showed good predictive efficacy for T1 stage HCC. The AUC value of SAA4 was 0.8064, which was also higher than that of AFP (AUC = 0.7048). Meanwhile, the combination of AFP and SAA4 also showed the best predictive efficacy for HCC with T1 stage (AUC = 0.8816). These results suggested that SAA4 was superior to AFP in predicting HCC and that SAA4 was a good complement for diagnosis of AFP-negative HCC.

## Discussion

Currently, HCC is one of the leading causes of tumor-related death. There are various treatment methods for HCC, such as surgical resection [19], radiofrequency ablation [20, 21], transcatheter arterial chemoembolization (TACE) [22], immunotherapy [23], targeted therapy [24], and liver transplantation [25, 26]. Among them, surgical resection is recommended for patients with single HCC tumors with good liver function and without portal hypertension [27]. However, at present, most patients are diagnosed at an advanced stage, at which point 5-year OS rate is less than 15%, while the rate for patients with early HCC can reach more than 70% [3]. In addition, the 5-year recurrence rate after tumor resection is over 50%, and regular follow-up monitoring after surgery is very important. Hence, early screening and effective postoperative surveillance can significantly improve the prognosis of tumor patients.

Currently, there are a variety of diagnostic methods for HCC, including serological alpha-fetoprotein detection, ultrasound, CT, MRI, and percutaneous liver biopsy [3, 28]. Among them, US and serological AFP detection are the main means for the early diagnosis and surveillance of HCC. However, both of these methods lack sufficient sensitivity and accuracy. Therefore, it is crucial to find a novel biomarker for the diagnosis of HCC, especially for early or recurrent HCC.

In this study, we investigated the expression changes of SAA4 during the progression of HCC and the predictive ability for HCC, even in the early stage. First, we compared the gene expression of SAA4 between tumor and paracancerous tissues. The results showed that unlike the traditional biomarkers, SAA4 was significantly downregulated in tumor tissues versus paracancerous tissues. In addition, the difference in the protein expression of SAA4 between tumor and paracancerous tissues was further validated, and the IHC results from the THPA and our center indicated that similar to the results for gene expression, the staining intensity of SAA4 protein in paracancerous tissues was significantly stronger than that in tumor tissues. Then, we found that SAA4 expression decreased with the progression and poor differentiation of HCC. However, there was no significant difference in the expression of SAA4 in different stages in the analysis of the N and M stages, possibly because of the limited sample size of patients in N1 and M1 stages. Combining the results of survival analysis based on the TCGA and K‒M plotter databases, we found that high SAA4 expression in tumor tissues was closely related to good outcomes in HCC. This indicates that SAA4 may play an indicator role in the survival of patients with HCC.

In addition, to further verify the predictive effect of SAA4 on HCC, ROC curve analysis was performed. We found that SAA4 had a very good predictive performance for HCC, even early-stage HCC and was superior to AFP. These results suggest that SAA4 is a promising biomarker for early HCC screening and can be used as a candidate marker for AFP-negative HCC screening. However, further studies on the predictive efficacy of serum SAA4 levels for HCC are needed to facilitate clinical applications.

To further identify the mechanism underlying the decreased expression of SAA4 in HCC, we conducted GSEA. We found that the expression of SAA4 is closely related to the complement and coagulation cascades, metabolism of substances such as fatty acids and amino acids, and the structure of extracellular matrix and its interaction with receptors.

In this study, complement and coagulation cascades pathway was enriched in the group with high expression of SAA4 in HCC. However, there is a lack of evidence regarding how SAA4 exerts its antitumor effects on HCC through the complement and coagulation cascades pathway, and further experimental studies are needed to explore the potential mechanisms.

In addition, this study also showed that SAA4 expression was positively related to the fatty acid metabolism pathway. There is evidence that abnormal fatty acid accumulation and inhibition of fatty acid oxidation are critical for HCC progression [29]. In this study, SAA4 expression was closely related to fatty acid metabolism, suggesting that SAA4 may inhibit the aggressiveness of HCC by promoting fatty acid oxidation.

The extracellular matrix (ECM) is secreted by cells as its structure and biochemical support and plays a vital role in cell proliferation, differentiation and microenvironment homeostasis [30]. The dysregulation of the ECM can significantly affect the progression of tumors. The results of GSEA showed that the expression of SAA4 was negatively correlated with the ECM receptor interaction pathway. We hypothesized that SAA4 may be involved in maintaining the balance of ECM structure and function and microenvironment homeostasis. SAA4 expression is reduced in HCC, resulting in an imbalance between the ECM and microenvironment homeostasis, which promotes the progression of HCC.

However, this study still had some limitations. First, although this study assessed the predictive effect of SAA4 gene expression on HCC, to facilitate the application in clinic, we still need to study the effectiveness of serum SAA4 protein levels in predicting HCC. Second, this study predicted the potential mechanisms of SAA4 involvement in HCC only through bioinformatics analysis, and we need to further verify the role of SAA4 through fundamental experiments in subsequent studies.

## Conclusions

In conclusion, this study revealed that the expression of SAA4 was low in HCC and had a decreasing trend during the progression of HCC. In addition, HCC patients with low expression of SAA4 had a worse prognosis. Our study also demonstrated that SAA4 may play a role as an indicator for HCC and a complementary role in the diagnosis of AFP-negative HCC.

## Supplementary Information

Below is the link to the electronic supplementary material.

Supplementary file1 (TIF 196 KB)

## Data Availability

The datasets used and/or analyzed during the current study are available from the corresponding author upon reasonable request.

## References

[1] Sung H, Ferlay J, Siegel RL, et al. Global Cancer Statistics 2020: GLOBOCAN Estimates of Incidence and Mortality Worldwide for 36 Cancers in 185 Countries. CA Cancer J Clin. 2021;71:209–49. 33538338 10.3322/caac.21660

[2] Cao M, Li H, Sun D, et al. Cancer burden of major cancers in China: A need for sustainable actions. Cancer Commun (Lond). 2020;40:205–10. 32359212 10.1002/cac2.12025 PMC7667573

[3] Wang W, Wei C. Advances in the early diagnosis of hepatocellular carcinoma. Genes Dis. 2020;7:308–19. 32884985 10.1016/j.gendis.2020.01.014 PMC7452544

[4] Marrero JA, Feng Z, Wang Y, et al. Alpha-fetoprotein, des-gamma carboxyprothrombin, and lectin-bound alpha-fetoprotein in early hepatocellular carcinoma. Gastroenterology. 2009;137:110–8. 19362088 10.1053/j.gastro.2009.04.005 PMC2704256

[5] Zhang B, Yang B. Combined alpha fetoprotein testing and ultrasonography as a screening test for primary liver cancer. J Med Screen. 1999;6:108–10. 10444731 10.1136/jms.6.2.108

[6] Tzartzeva K, Obi J, Rich NE, et al. Surveillance Imaging and Alpha Fetoprotein for Early Detection of Hepatocellular Carcinoma in Patients With Cirrhosis: A Meta-analysis. Gastroenterology. 2018;154(1706–18): e1. 10.1053/j.gastro.2018.01.064 PMC5927818 29425931

[7] Singal A, Volk ML, Waljee A, et al. Meta-analysis: surveillance with ultrasound for early-stage hepatocellular carcinoma in patients with cirrhosis. Aliment Pharmacol Ther. 2009;30:37–47. 19392863 10.1111/j.1365-2036.2009.04014.x PMC6871653

[8] Colli A, Fraquelli M, Casazza G, et al. Accuracy of ultrasonography, spiral CT, magnetic resonance, and alpha-fetoprotein in diagnosing hepatocellular carcinoma: a systematic review. Am J Gastroenterol. 2006;101:513–23. 16542288 10.1111/j.1572-0241.2006.00467.x

[9] Hanna RF, Miloushev VZ, Tang A, et al. Comparative 13-year meta-analysis of the sensitivity and positive predictive value of ultrasound, CT, and MRI for detecting hepatocellular carcinoma. Abdom Radiol (NY). 2016;41:71–90. 26830614 10.1007/s00261-015-0592-8

[10] Kim SY, An J, Lim YS, et al. MRI With Liver-Specific Contrast for Surveillance of Patients With Cirrhosis at High Risk of Hepatocellular Carcinoma. JAMA Oncol. 2017;3:456–63. 27657493 10.1001/jamaoncol.2016.3147 PMC5470420

[11] Sun L, Ye RD. Serum amyloid A1: Structure, function and gene polymorphism. Gene. 2016;583:48–57. 26945629 10.1016/j.gene.2016.02.044 PMC5683722

[12] Kluve-Beckerman B, Drumm ML, Benson MD. Nonexpression of the human serum amyloid A three (SAA3) gene. DNA Cell Biol. 1991;10:651–61. 1755958 10.1089/dna.1991.10.651

[13] Ducret A, Bruun CF, Bures EJ, et al. Characterization of human serum amyloid A protein isoforms separated by two-dimensional electrophoresis by liquid chromatography/electrospray ionization tandem mass spectrometry. Electrophoresis. 1996;17:866–76. 8783012 10.1002/elps.1150170508

[14] de Beer MC, Yuan T, Kindy MS, et al. Characterization of constitutive human serum amyloid A protein (SAA4) as an apolipoprotein. J Lipid Res. 1995;36:526–34. 7775864

[15] De Buck M, Gouwy M, Wang JM, et al. Structure and Expression of Different Serum Amyloid A (SAA) Variants and their Concentration-Dependent Functions During Host Insults. Curr Med Chem. 2016;23:1725–55. 27087246 10.2174/0929867323666160418114600 PMC5405626

[16] Malle E, De Beer FC. Human serum amyloid A (SAA) protein: a prominent acute-phase reactant for clinical practice. Eur J Clin Invest. 1996;26:427–35. 8817153 10.1046/j.1365-2362.1996.159291.x

[17] Tamamoto T, Ohno K, Ohmi A, et al. Verification of measurement of the feline serum amyloid A (SAA) concentration by human SAA turbidimetric immunoassay and its clinical application. J Vet Med Sci. 2008;70:1247–52. 19057145 10.1292/jvms.70.1247

[18] Diakos CI, Charles KA, McMillan DC, et al. Cancer-related inflammation and treatment effectiveness. Lancet Oncol. 2014;15:e493-503. 25281468 10.1016/S1470-2045(14)70263-3

[19] Bruix J, Sherman M. Practice Guidelines Committee AAftSoLD. Management of hepatocellular carcinoma Hepatology. 2005;42:1208–36. 16250051 10.1002/hep.20933

[20] Breen DJ, Lencioni R. Image-guided ablation of primary liver and renal tumours. Nat Rev Clin Oncol. 2015;12:175–86. 25601446 10.1038/nrclinonc.2014.237

[21] Xu XL, Liu XD, Liang M, et al. Radiofrequency Ablation versus Hepatic Resection for Small Hepatocellular Carcinoma: Systematic Review of Randomized Controlled Trials with Meta-Analysis and Trial Sequential Analysis. Radiology. 2018;287:461–72. 29135366 10.1148/radiol.2017162756

[22] Brown KT, Do RK, Gonen M, et al. Randomized Trial of Hepatic Artery Embolization for Hepatocellular Carcinoma Using Doxorubicin-Eluting Microspheres Compared With Embolization With Microspheres Alone. J Clin Oncol. 2016;34:2046–53. 26834067 10.1200/JCO.2015.64.0821 PMC4966514

[23] Yau T, Park JW, Finn RS, et al. Nivolumab versus sorafenib in advanced hepatocellular carcinoma (CheckMate 459): a randomised, multicentre, open-label, phase 3 trial. Lancet Oncol. 2022;23:77–90. 34914889 10.1016/S1470-2045(21)00604-5

[24] Meyer T, Fox R, Ma YT, et al. Sorafenib in combination with transarterial chemoembolisation in patients with unresectable hepatocellular carcinoma (TACE 2): a randomised placebo-controlled, double-blind, phase 3 trial. Lancet Gastroenterol Hepatol. 2017;2:565–75. 28648803 10.1016/S2468-1253(17)30156-5

[25] Mazzaferro V, Regalia E, Doci R, et al. Liver transplantation for the treatment of small hepatocellular carcinomas in patients with cirrhosis. N Engl J Med. 1996;334:693–9. 8594428 10.1056/NEJM199603143341104

[26] Sapisochin G, Bruix J. Liver transplantation for hepatocellular carcinoma: outcomes and novel surgical approaches. Nat Rev Gastroenterol Hepatol. 2017;14:203–17. 28053342 10.1038/nrgastro.2016.193

[27] Tabrizian P, Jibara G, Shrager B, et al. Recurrence of hepatocellular cancer after resection: patterns, treatments, and prognosis. Ann Surg. 2015;261:947–55. 25010665 10.1097/SLA.0000000000000710

[28] Forner A, Reig M, Bruix J. Hepatocellular carcinoma. Lancet. 2018;391:1301–14. 29307467 10.1016/S0140-6736(18)30010-2

[29] Ma APY, Yeung CLS, Tey SK, et al. Suppression of ACADM-mediated fatty acid oxidation promotes hepatocellular carcinoma via aberrant CAV1/SREBP1 signaling. Cancer Res. 2021;81:3679–92. 33975883 10.1158/0008-5472.CAN-20-3944

[30] Biteau B, Hochmuth CE, Jasper H. Maintaining tissue homeostasis: dynamic control of somatic stem cell activity. Cell Stem Cell. 2011;9:402–11. 22056138 10.1016/j.stem.2011.10.004 PMC3212030

